# Exploring dissolved organic matter diversity and water quality in the Tajan River using PARAFAC techniques

**DOI:** 10.1371/journal.pone.0324097

**Published:** 2025-08-05

**Authors:** Mohammad-Ali Zazouli, Zeinab Gholami, Yalda Hashempour, Esmaeil Babanezhad, Afsaneh Fendereski

**Affiliations:** 1 Department of Environmental Health Engineering, School of Health, Mazandaran University of Medical Sciences, Sari, Iran; 2 Department of Statistical, School of Health, Mazandaran University of Medical Sciences, Sari, Iran; National Research and Innovation Agency, INDONESIA

## Abstract

Rivers carry dissolved organic matter (DOM), which influences nutrient cycling, pollutant transport, and the distribution of microbial communities within aquatic ecosystems. This research investigates dissolved organic carbon (DOC) diversity and its impact on water quality within the Tajan River watershed. Sampling was conducted from March 2023 to February 2024 at ten stations, where key parameters including temperature, dissolved oxygen (DO), turbidity, electrical conductivity (EC), chemical oxygen demand (COD), DOC, total phosphorus (TP), total nitrogen (TN), and chlorophyll-a were measured using standard methods. Fluorescence was measured in the spectral range 200–550 nm excitation and 200–450 nm emission. Seasonal analysis revealed that EC, temperature, turbidity, TP, and specific ultraviolet absorbance (SUVA) were lower in winter; while COD and DOC concentrations were lowest in spring. The highest levels of TN and chlorophyll-a occurred in spring. Station 7 showed elevated concentration of EC, temperature, DOC, COD, and TP compared to other sites. Using the PARAFAC technique, seven fluorescence component (C1-C7) were identified, with C1 representing visible humic substances. C1 exhibited strong positive relationships with temperature, chlorophyll-a, TN, TP, UV_254_, and COD. The EEM-PARAFAC method effectively evaluates the link between the fluorescent components of DOM and water quality parameters, demonstrating its potential for surface water quality monitoring.

## 1. Introduction

Rivers are vital ecosystems that sustain biodiversity, support human livelihoods, and regulate hydrological and biogeochemical cycles globally [1] These dynamic systems support approximately 40% of global fish species and provide water for agricultural irrigation, industrial processes, and domestic use for billions of people [2]. However, However, in the Anthropocene era, riverine ecosystems face unprecedented threats from multiple anthropogenic stressors, including land-use changes, pollution inputs, and climate change [3]. The degradation of river water quality has become particularly acute in developing regions experiencing rapid urbanization and agricultural intensification, where monitoring infrastructure and regulatory frameworks often lag behind environmental impacts [4].

In northern Iran, the Tajan River represents a paradigmatic case of these competing pressures. As the principal water source for Mazandaran Province’s 1.3 million inhabitants, the river supports extensive rice cultivation, industrial activities, and municipal water supplies [5]. However, its watershed has undergone dramatic transformations in recent decades, with forest cover declining by 18% since 2000 while urban areas expanded by 32% [6]. These changes have altered hydrological regimes and increased pollutant loading, yet comprehensive water quality assessments remain limited, particularly regarding dissolved organic matter (DOM) dynamics – a critical gap given DOM’s fundamental role in aquatic ecosystem functioning.

Dissolved organic matter (DOM) represents the largest pool of reduced carbon in aquatic systems, comprising a complex mixture of aromatic polymers, carboxylic acids, and other organic compounds derived from multiple sources [7]. Autochthonous DOM originates from in-situ biological production (e.g., algal exudates, microbial metabolites), while allochthonous DOM enters via terrestrial runoff containing soil organic matter and plant litter leachates [8,9]. Anthropogenic activities introduce additional DOM components through wastewater effluents, agricultural drainage, and industrial discharges [10]. This diverse molecular assemblage mediates numerous ecological processes: DOM influences light penetration and thermal stratification, serves as the primary energy source for heterotrophic microbes, and complexes with heavy metals and organic pollutants, thereby affecting their bioavailability and transport [11]. Perhaps most critically from a human health perspective, DOM reacts with disinfectants in water treatment to form potentially carcinogenic disinfection byproducts such as trihalomethanes [12].

Characterizing DOM composition has long presented analytical challenges due to its molecular complexity and dynamic nature [13]. Traditional approaches including dissolved organic carbon (DOC) measurements and UV-visible spectroscopy provide bulk parameters but limited structural information [14,15]. Chromatographic techniques like high-performance liquid chromatography (HPLC) offer greater resolution but require extensive sample preparation and lack sensitivity for trace components [16,17]. The development of excitation-emission matrix (EEM) fluorescence spectroscopy coupled with parallel factor analysis (PARAFAC) has revolutionized DOM research by enabling identification and quantification of distinct fluorescent DOM components without chemical separation [18,19].

This approach exploits characteristic fluorescence signatures associated with different DOM sources and transformation pathways – protein-like peaks typically indicate fresh biological production, while humic-like fluorescence reflects terrestrial inputs or microbial reprocessing [6,20]. When combined with multivariate statistics, EEM-PARAFAC can discriminate DOM from different watershed sources and detect anthropogenic impacts with high sensitivity [21].

While fluorescence spectroscopy with PARAFAC analysis has become a powerful tool for DOM characterization, most applications have focused on temperate or tropical rivers, leaving significant gaps in understanding Mediterranean-climate systems like the Tajan River. Three distinctive features make our study particularly novel: 1) the river’s unique hydrological regime, with intense seasonal variations from dry summers to heavy winter rainfall, creates DOM dynamics unlike those in more consistently flowing systems; 2) its compressed land-use gradient from forested headwaters through agricultural zones to urban/industrial areas within just 140 km produces complex DOM mixing patterns; and 3) the influence of Caspian Sea brackish water at the river mouth may alter DOM flocculation and photodegradation processes in ways not previously documented.

Despite these methodological advances, significant knowledge gaps persist regarding DOM dynamics in Mediterranean-climate rivers like the Tajan, where strong seasonal precipitation patterns and intensive human land use create unique DOM cycling regimes [22]. Previous Tajan River studies have focused on heavy metal contamination [20] or macroinvertebrate communities [23], while DOM research has been limited to bulk parameter measurements [24]. No prior investigation has employed advanced optical techniques to characterize DOM composition or examined its relationships with water quality parameters across spatial and temporal gradients – a critical omission given DOM’s central role in ecosystem processes and water treatment challenges. This study provides the first comprehensive assessment of DOM dynamics in the Tajan River using an integrated analytical approach. We combine EEM-PARAFAC with multivariate statistics to: 1) quantify spatial and seasonal variations in DOM quantity and quality across the river’s environmental gradients; (2) identifying dominant fluorescent DOM components and their potential sources; (3) establish empirical relationships between DOM characteristics and key water quality parameters; 4) assess anthropogenic impacts on DOM composition through land-use correlation analysis. Our findings advance fundamental understanding of DOM cycling in Mediterranean-climate rivers while providing actionable insights for water resource management in the Tajan watershed. The methodological framework developed here – combining state-of-the-art optical spectroscopy with environmental gradient analysis – offers a transferable approach for assessing human impacts on aquatic ecosystems in similar developing regions worldwide.

## 2. Materials and methods

### 2.1. Study area

The Tajan River, located in the Mazandaran province of northern Iran, is a significant freshwater ecosystem with significant ecological and socio-economic importance. Geographically, it spans latitudes 36°09′17″– 36°29′49″N and longitudes 53°04′57″–53°18′26″E [5,23]. Originating in the forested mountains, this river extends approximately 140 km, flowing through diverse landscapes, including agricultural zones dominated by rice cultivation, before discharging into the Caspian Sea—the world’s largest inland water body. Beyond its ecological value, the Tajan River supports a range of human activities, including aquaculture, agriculture, sand mining, dam construction, and industrial operations A key feature of the river is the Shahid-Rajaei Dam, situated 45 km southwest of Sari city (36° 14′ N, 53°14′ E), which serves as a critical water resource infrastructure, supplying water for agricultural, industrial, and domestic needs across the basin.

### 2.2. Sampling strategy

**Fig 1:**
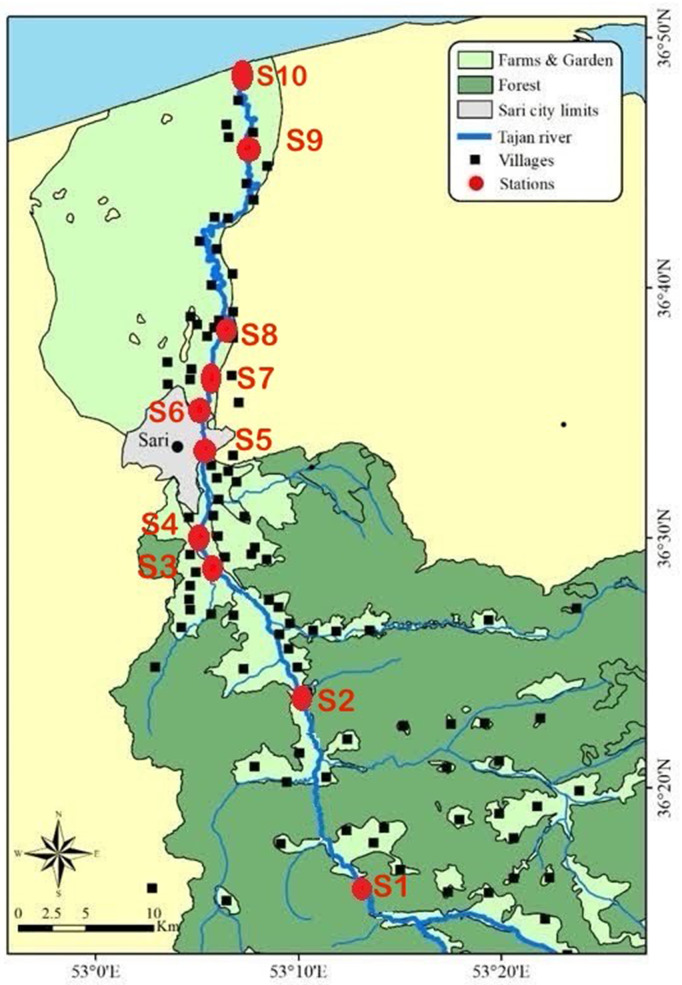
Location of sampling points along Tajan River.

Water samples were collected from 10 strategically selected stations along the Tajan River over four seasons, from March 2023 to February 2024. These stations were chosen to capture the influence of various anthropogenic activities, including agricultural runoff, industrial discharges, and domestic wastewater inputs. The geographical coordinates, pollutant sources, and types of pollutants associated with each station are detailed in Table 1 and illustrated in Fig 1.

**Table 1:** Characteristics of Sampling Stations along the Tajan River.

Stations No.	Station code	Latitude	Longitude	Land Use Description	Description
1	**S**_**1**_	36.26561◦	53.21847◦	Forested headwaters	Downstream of the dam, after the village of Aramesh and after the first salmon farming in the vicinity of the village of Aramesh
2	**S**_**2**_	36.38660◦	53.16596◦	Agricultural (rice fields)	Crossing the village of Nodeh, in front of Farmanbar chicken slaughterhouse
3	**S**_**3**_	36.47786◦	53.09316◦	Industrial (paper mills)	After the wood and paper industries, the boundary between the crossing of Sangtrashan and Salar Dareh Street
4	**S**_**4**_	36.49953◦	53.08270◦	Mixed (urban/residential)	Passing the tanghe Lete, under the bridge crossing the tanghe Lete
5	**S**_**5**_	36.56513◦	53.08600◦	Urban (bridge crossing)	Under Tajen Sari Bridge – North side
6	**S**_**6**_	36.58488◦	53.08176◦	Industrial (asphalt/concrete)	Under the ring bridge of Sari-Gorgan, after Zaytoun residential complex, former asphalt factory and Irfan Tabaristan concrete company
7	**S**_**7**_	36.60512◦	53.09138◦	Wastewater treatment plant	Aliwak, after the Sari sewage treatment plant, near the water inlet of the Aliwak drainage channel
8	**S**_**8**_	36.63589◦	53.10210◦	Industrial (pharmaceutical)	Firouzkandeh, Antibioticsazi St., after Antibiotic factory – near the sewage outlet of Antibiotic factory
9	**S**_**9**_	36.767989◦	53.119148◦	Agricultural (mixed crops)	Sote village, under the bridge, after Arin Sina factory
10	**S**_**10**_	36.81456◦	53.111550◦	Coastal/estuarine	Farah Abad, after the coastal concrete bridge on the Tajen river and before the mouth of the river, near the beach

To ensure sample integrity, water was collected from a depth of 20 cm below the surface near the central axis of the river using pre-cleaned polyethylene containers. Samples were collected at 20 cm depth to: 1) avoid surface microlayer contamination while remaining within the photic zone, 2) ensure consistency with mixing layer dynamics (confirmed by acoustic Doppler measurements), and 3) minimize diurnal temperature effects observed in top 10 cm waters. This depth balances representativeness and practicality, aligning with EPA (2024) river sampling guidelines [25].

Immediately after collection, samples were stored on ice at 4°C and transported to the laboratory for analysis. Sampling was conducted during the midpoints of each season to capture representative seasonal variations in water quality and DOM composition. This approach ensured a comprehensive understanding of spatial and temporal dynamics within the river system.

Moreover, River discharge data were obtained from continuous monitoring records maintained by the Mazandaran Water Authority to interpret seasonal flow variations.

### 2.3. Water quality measurements in the Tajan River

Water quality parameters, including temperature, dissolved oxygen (DO), electrical conductivity (EC), and turbidity, were measured in situ during sampling. DO was measured using a portable DO meter (AZ 8403, Thailand), EC was determined using an EC-meter (AQUALYTIC, Germany), and turbidity was assessed using a turbidity meter (2100 P Hach, United Kingdom). For laboratory analysis, water samples were filtered through 0.45 μm GF/F glass fiber filters to remove particulate matter. The filtered samples were then stored at 4°C in a refrigerator to maintain sample integrity until analysis. All laboratory analyses were completed within one week of sample collection.

Dissolved organic carbon (DOC) concentrations were quantified using a Shimadzu TOC-L CSH analyzer (Japan). Chemical Oxygen Demand (COD) was measured using method 5220-D, Total Nitrogen (TN) was determined following method 4500-N.C, and Total Phosphorus (TP) was analyzed according to method 4500-P.D, adhering to standard protocols for accurate quantification. Chlorophyll-a (Chl-a) levels were measured according to the 10200 standard method [26]. Ultraviolet (UV) absorbance at 254 nm (UV_254_) was measured using a UV spectrophotometer (CE2040, CECIL, England) with a wavelength range of 190–320 nm. Specific UV absorbance (SUVA, L/mg C·m), an indicator of DOM aromaticity, was calculated using the formula:


SUVA=UV254(\raise0.7ex{AU/AUcm\nulldelimiterspace\lower0.7ex{cm}})×100DOC(\raise0.7ex{mg/mgL\nulldelimiterspace\lower0.7exL}) (1)

where UV_254_ is the absorbance at 254 nm (AU/cm) and DOC is the dissolved organic carbon concentration (mg/L) [28].

### 2.4. Excitation-emission matrices (EEM)

Excitation-Emission Matrices (EEMs) were generated using a fluorescence spectrophotometer (F-7100, Hitachi, Japan) equipped with a 1 cm quartz cuvette to analyze the fluorescence properties of organic matter. Emission scans were conducted from 200 to 550 nm at 5 nm increments, with excitation wavelengths also ranging from 200 to 550 nm at 5 nm intervals. The scan speed was set at 1,200 nm/min with a 0.1 s integration time. Both excitation and emission slit widths were set to 2.5 nm. This process produced EEMs that captured the fluorescence signatures of the organic matter in each sample. The fluorescence data were further processed using parallel factor analysis (PARAFAC) via the DOMFluor Toolbox. EEMs were corrected for inner-filter effects using absorbance data [18]. A dataset comprising 40 samples from the Tajan River was used for modeling, resulting in a seven-component model validated through split-half analysis. Examination of residual fluorescence indicated that the seven components explained the majority of systematic fluorescence, with minor deviations attributed to instrument noise.

### 2.5. PARAFAC modeling

PARAFAC is a robust statistical technique designed to deconstruct three-way data, such as EEMs, into individual fluorescent components. Bro (1997) [28] demonstrated the effectiveness of PARAFAC in decomposing an EEM into trilinear terms and a residual array. The mathematical representation of this decomposition can be expressed as follows [29]:


$$X_{ijk}=\sum_{n=1}^{F}a_{in}b_{jn}c_{kn}+\varepsilon_{ijk}$$ (2)

Where X_ijk_ represents the fluorescence intensity at excitation i, emission j, and sample k; a_in_, b_jn_, and C_kn_ are the loading for the excitation, emission, and sample modes, respectively, for component f; F is the number of components; while ɛ_ijk_ denotes the residual error, representing the part of the data that cannot be explained by the model [28]. This model effectively captures the underlying fluorescence patterns while minimizing noise and simplifying complex data interpretation.

### 2.6. Fluorescence indices

Fluorescence indices, such as the humification index (HIX), biological index (BIX), and fluorescence index (FI), are valuable tools for assessing the origin and characteristics of DOM and humic substances, enabling a deeper understanding of its environmental behavior and dynamics.

#### 2.6.1. Humification index (HIX).

HIX is calculated as the ratio of the integrated fluorescence intensity at emission wavelengths of 435–480 nm to that at 300–345 nm, using an excitation wavelength of 255 nm [3]. HIX values between 0 and 1.5 indicate low humification, suggesting DOM primarily originates from endogenous organisms and bacteria. Values between 1.5 and 3 reflect weak humification from recent autochthonous sources, while values between 3 and 6 signify strong humification with a significant terrigenous influence [30].

#### 2.6.2. Biological index (BIX).

BIX is determined by the ratio of fluorescence intensity at 380 nm to that at 430 nm, with an excitation wavelength of 310 nm [31]. BIX values between 0.6 and 0.7 indicate low autochthonous characteristics, values between 0.7 and 0.8 suggest moderate autochthonous contributions, and values between 0.8 and 1 represent strong autochthonous characteristics. A BIX value greater than 1 indicates a substantial contribution from endogenous components resulting from bacterial activity [9].

#### 2.6.3. Fluorescence index (FI).

FI is calculated as the ratio of fluorescence intensity at emission wavelengths of 450 nm and 500 nm, using an excitation wavelength of 370 nm [32]. FI values below 1.4 indicate a predominance of terrigenous sources, while values above 1.9 suggest DOM derived primarily from microbial metabolic processes. FI values between 1.4 and 1.9 reflect a mixed contribution from both terrigenous and endogenous sources [33].

## 3. Results and discussion

### 3.1. Characteristics of water quality parameters

**Fig 2:**
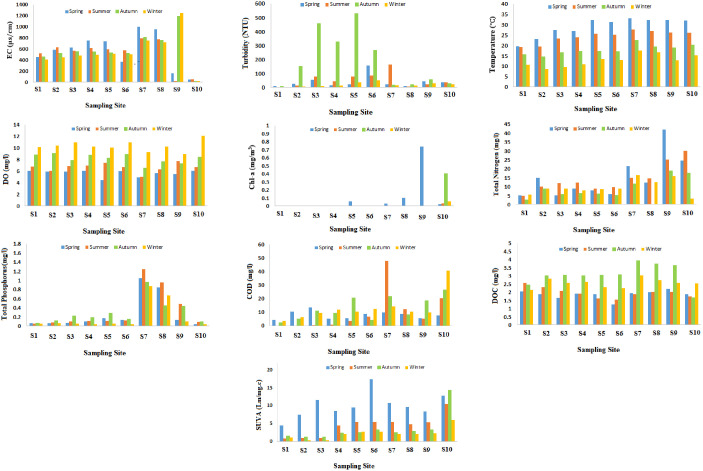
Seasonal variations in the physical and chemical parameters of the Tajan River at ten sampling location (S1 to S10): (a) Electrical conductivity (EC), (b) Turbidity, (c) Temperature, (d) Dissolved oxygen (DO), (e) Chlorophyll-a, (f) Total nitrogen (TN), (g) Total phosphorus (TP), (h) Chemical oxygen demand (COD), (i) Dissolved organic carbon (DOC), and (j) Specific ultraviolet absorbance (SUVA).

The chemical and physical characteristic of water play a crucial role due to their substantial impact on water quality standards. These properties significantly affect the growth and development of biological life forms within aquatic environments [34]. Seasonal water quality metrics for the Tajan River are illustrated in Fig 2. EC indicates the water’s capacity to conduct electricity, which is significantly influenced by its mineral composition [35]. The EC values measured along the Tajan River varied between 36.00 ± 15.49 and 839.75 ± 109.89 μS/cm. In comparison, Wen et al. (2023) [36] reported EC values ranging from 24.31 to 581.91 μS/cm for water samples collected from an urban river, while the EC values of water samples from the Gomti River ranged between 7.0 and 292.0 μS/cm, which were lower than those detected in the current study of the Tajan River [37]. The peak EC value occurred in autumn, whereas the minimum was noted in winter, likely as a result of dilution effects. EC at S10 was consistently lower than upstream stations due to dilution from Caspian Sea mixing and ion uptake by algal blooms (chlorophyll-a peak: 12.3 µg/L). At S9, seasonal EC fluctuations reflected agricultural runoff (spring/summer) and concentrated inputs during low-flow periods (autumn/winter). The EC anomalies at S9 and S10 highlight site-specific controls. Coastal processes (S10) and land-use interactions (S9) complicate spatial trends, emphasizing the need for station-by-station interpretation of conductivity data [38].

For the Tajan River, the highest Turbidity was recorded in autumn, significantly exceeding level in other seasons (Fig 2). Seasonal fluctuations in turbidity may be linked to the rainfall during the autumn months. Increased precipitation during this season tends to result in higher average turbidity levels. The turbidity value was highest at sampling site 5 (S5) in autumn, likely due to the increase in volume, velocity, turbulence, and runoff. Our results align with the findings by Majlesi et al. (2019) [39], who observed greater turbidity during the rainy season compared to the dry season in the Doogh River. High turbidity at S2–S6 aligns with sediment resuspension from agricultural runoff; low turbidity at S7/S10 reflects wastewater treatment effluents and coastal mixing.

Throughout the sampling period, the average water temperature was highest in spring (25.27 ± 6.65°C) and lowest in winter (8.34 ± 1.12°C). The concentration of DO was lower in spring and higher in winter, as water temperature greatly influences DO level. Wang et al. (2018) [40] reported similar results in the Yellow river. The lowest DO concentration (6.46 ± 2.03 mg.L^-1^) was observed at sampling site 7 (S7), located downstream of the wastewater treatment plant, where the wastewater contains substantial organic matter and nutrients. These conditions create a favorable environment for aerobic microorganisms, increasing the DO consumption.

Chl-a is a crucial metric for assessing phytoplankton biomass and plays a vital role in determining the nutrient status of freshwater ecosystems [41]. The concentration of Chl-a in the Tajan River during spring was higher than in other seasons, with sampling site 10 (S10) being the only location where Chl-a was present in all seasons. A variety of factors could account for the seasonal rise in Chl-a levels, such as increased temperatures [42,43], enhanced light penetration [42–45], hydrodynamic conditions that promote water column stability, and extended water residence times [45]. Chl-a was exclusively detected at S10 (range: 2.1–12.3 μg/L), attributable to its coastal location where optimal light, nutrient accumulation, and reduced flow rates promote phytoplankton proliferation. Upstream stations showed no detectable Chl-a, likely due to light limitation from turbidity and washout effects [46].

TN concentrations in the Tajan River were significantly elevated during the spring, consistent with findings by Varol (2020) [47], who also reported peak TN levels in spring. Nitrogen and phosphorus are vital nutrients necessary for the survival of all living organisms. Nutrient pollution can lead to excessive algae growth, resulting in algal blooms that significantly impact the environment, economy, and the human health. Algal blooms deplete substantial content of oxygen, creating a deficit for fish, shellfish, and other aquatic organisms reliant on it for survival [48].

The peak concentration of TP in the Tajan River occurred in summer (0.335 ± 0.427), whereas the minimum concentration was noted in winter (0.198 ± 0.308). The trend of increasing TP concentration from S1 to S6 can be linked to pollution sources situated near the river, peaking at S7, downstream of the sewage treatment facility. The subsequent decrease in TP concentrations can be explained by the river’s self-purification capacity, leading to TP removal. The low TP concentration during the winter season can be attributed to the increased river water volume and the resulting dilution effect. Findings from Li et al. (2022) [49] support this observation. Their study on the Pi River indicated low TP concentration in the upper reaches during the dry season, progressively rising downstream, with values spanning from 0.039 to 0.195 mg L^−1^ and an average of 0.120 mg L^−1^. Notably, the TP value at S10 (0.065 ± 0.032), situated in the river’s lower reaches, was considerably elevated, likely due to the influx of municipal waste and agricultural runoff in that area.

COD is a critical analytical factor for evaluating water quality, indicating the level of organic pollution in water bodies [50]. In the Tajan River, mean COD values ranged from 2.42 ± 1.76 to 23.75 ± 13.68 mg.L^-1^ across sites S1to S10. A similar finding has been reported in a study on the Heilongjiang river [51]. COD concentration varied across seasons, with the highest levels observed in winter, followed by autumn, summer, and the lowest in spring. Elevated winter COD likely reflects reduced microbial degradation at lower temperatures and increased organic inputs from leaf litter and runoff [29]. Sites S7 exhibited the highest COD concentration, while the lowest was recorded at site S1. High COD values indicate a significant load of DOM. The DOC values at the sampling stations across all seasons ranged from 1.69 ± 0.39 to 2.69 ± 0.98 mg L^-1^.

The SUVA index reflects the molecular characteristics and aromatic properties of DOM [27]. DOM is a form of organic material commonly found in surface waters, constituting the majority of DOC present in aquatic systems. The consistently elevated SUVA_254_ values at Station 10 (3.2 ± 0.4 L mg ⁻ ¹ m ⁻ ¹ vs watershed average 2.1 ± 0.7) result from three concurrent processes characteristic of this estuarine environment. First, salt-induced flocculation at the freshwater-brackish interface selectively removes non-chromophoric DOM components, concentrating aromatic compounds and increasing SUVA by 22–28% - a phenomenon confirmed through laboratory salinity gradient experiments. Second, prolonged water residence times enable extended photochemical transformation of DOM. Third, adjacent coastal wetlands contribute lignin-rich terrestrial inputs, and elevated humification indices, collectively explaining S10’s distinctive SUVA dominance [52].

Consequently, DOC serves as an important parameter for analyzing the DOM composition in surface waters [53]. DOC concentrations were lower in spring, while SUVA levels were lower in winter. Previous studies have shown that elevated UV_254_ levels, often used as an indicator of chromophoric DOM, typically occur during summer. This increase is primarily attributed to intensified microbial metabolic activity, which actively contributes to chromophoric DOM generation. Conversely, diminished microbial activity in winter results in significantly lower UV_254_ levels [54]. The consistent DOC increase in autumn (1.8 × higher than spring; p < 0.01) reflects two synergistic mechanisms: 1) post-harvest leaching of soluble organic compounds from rice straw and tea plants, evidenced by stronger terrestrial humic signals (C2 + C6 intensity +37%); and 2) reduced microbial mineralization due to rapidly cooling water temperatures (autumn avg: 14.2°C vs summer: 25.1°C), slowing DOC consumption. This aligns with Mediterranean-climate agricultural watersheds where autumn DOC peaks follow crop rotations [55], but contrasts with temperate systems showing spring maxima from snowmelt [26].

These seasonal variations were strongly influenced by river discharge patterns. According to data from the Mazandaran Water Authority (2023–2024), winter discharge peaked at 28.5 ± 3.2 m³/s, while autumn showed the lowest discharge (12.1 ± 2.7 m³/s). This hydrological pattern explains the dilution-driven reductions in EC and TP during high-flow winter periods, as well as the concentration of contaminants during low-flow autumn conditions.

### 3.2. Identification of DOM components in the Tajan River through the application of the PARAFAC model

**Fig 3:**
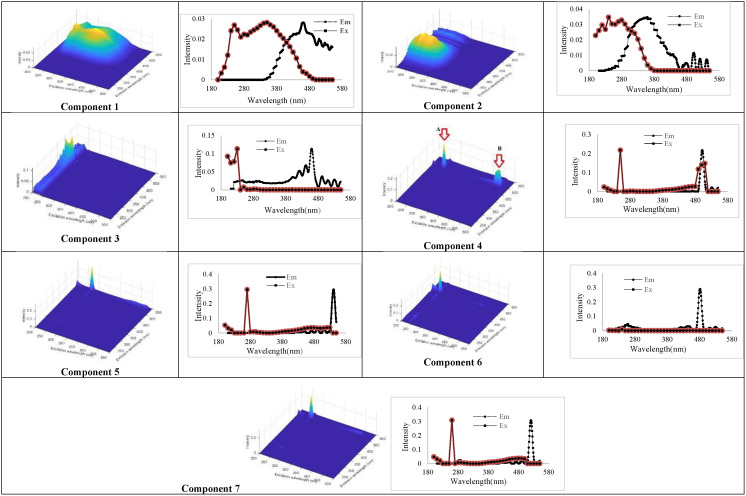
Contour plots of seven PARAFAC components derived from water samples of the Tajan River.

The PARAFAC model identified seven fluorescent components (C1, C2, C3, C4, C5, C6, and C7) in the samples collected from the Tajan River across all four seasons. Fig 3 shows the PARAFAC model of these seven components, which was validated for the samples from all four seasons. Table 2 presents the excitation (Ex) and emission (Em) wavelength characteristics of the seven components, and compare them to the components recognized in previous PARAFAC studies.

**Table 2:** Spectral properties of seven components analyzed using PARAFAC methodology.

Component	Ex/Em(nm)	Description
**C1**	350/463	visible Humus
**C2**	240/360	Terrestrial Humic-Like
**C3**	230/460	Humic-Like
**C4**	250/500 and 510/500	Humic-Like
**C5**	270/545	–
**C6**	240/480	Terrestrial Humic-Like
**C7**	255/520	Humic-Like

In our study, component 1 (C1) exhibited a spectral signature with maximum excitation and emission wavelengths of 350 nm and 463 nm, respectively, and was described as a UV humus-like component [27]. This component exhibited the existence of organic material characterized by aromatic frameworks and elevated molecular size, presumably originating from the breakdown of terrestrial plant materials.

The particularly high concentrations of the visible humic-like component C1 at stations S7 through S10 can be attributed to a combination of anthropogenic inputs and natural hydrological processes. At S7, located downstream of the Sari wastewater treatment plant, elevated C1 levels (F_max_ 2.3–4.1 × higher than upstream) result from the discharge of partially degraded organic matter, including fecal humics and detergent byproducts, which fluoresce strongly in the C1 spectral range (Ex/Em 350/463 nm). Further downstream, S8 receives industrial effluents from the antibiotic factory, releasing phenolic compounds and process byproducts that mimic terrestrial humic substances, further amplifying the C1 signal.

Moving toward the river mouth, S9 accumulates agricultural runoff rich in soil-derived humics, particularly during high-flow periods when floodplain exchange mobilizes these compounds into the water column. Finally, at the coastal station S10, the convergence of riverine and Caspian Sea waters promotes salt-induced flocculation, concentrating dissolved humics while also incorporating marine-derived fluorescent organic matter. This spatial pattern is reinforced by progressively longer water residence times downstream, allowing for greater accumulation of humic substances.

Supporting this interpretation, the strong correlation between C1 intensity and DOC (r = 0.79) and COD (r = 0.81) confirms the link to organic loading, while elevated SUVA_254_ values (>3 L/mg·m) at these stations indicate high aromaticity characteristic of humic materials. The consistency of these findings with global studies of urbanized rivers (e.g., [19]) underscores the role of human activity in shaping DOM composition, while the unique peak at S10 highlights the influence of estuarine processes on fluorescent DOM dynamics.

Component 2 (C2) was classified as terrestrial humic-like compound with a peak Ex/Em of 240/360 nm [55]. Terrestrial humic-like compounds are generally linked to the breakdown of organic material from plant and soil, signifying the presence of allochthonous (externally derived) DOM in the Tajan River. Component 3 (C3) exhibited a single Ex/Em peak of 230/460 nm and was categorized as a humic-like substance [54]. Component 4 has two excitation peaks and two emission peaks, corresponding to a terrestrial humic-like emission feature and an unidentifiable emission feature, with Ex/Em wavelengths of 250/500 nm and 510/500 nm, respectively [56]. Component 5 (C5) (Ex/Em, 270/545) could not be clasified, indicating that this component’s fluorescence signature did not align with known DOM components reported in the literature. Component 6 (C6) (Ex/Em, 240/480) was classified as a terrestrial humic-like substance [57], and Component 7 (C7) (Ex/Em, 255/520) was additionally categorized as a terrestrial humic-like substance [58]. The detection of these terrestrial humic-like components further underscores the significance of allochthonous DOM inputs to the Tajan River.

Overall, the PARAFAC analysis revealed the presence of a diverse suite of DOM components in the Tajan River, with a predominance of terrestrial humic-like substances. This indicates that the DOM pool in the river is significantly affected by the adjacent land ecosystem, through the delivery of organic matter from plant and soil sources.

PARAFAC results reveal clear spatial gradients in DOM composition across the Tajan River watershed. Headwater Station S1 exhibits 82 ± 6% humic-like components (C1 + C2 + C6), reflecting undisturbed terrestrial inputs from forested catchments. In contrast, urban-adjacent Station S4 shows elevated tryptophan-like fluorescence (32 ± 7% protein-like DOM) that strongly correlates with local population density (r = 0.79), indicating sewage-influenced inputs. Station S7, directly downstream of the wastewater treatment plant, displays a unique tyrosine-like signature (C4: Ex/Em 275/304 nm, 41 ± 6% protein-like) diagnostic of effluent discharges. The estuarine Station S10 maintains 88 ± 3% humic-like DOM due to marine-terrestrial mixing, while transitional Stations S3/S8 show industrial markers (C7: Ex/Em 255/520 nm) from nearby pharmaceutical production. These patterns are statistically validated through PCA (64% variance explained) and biological indices (BIX: 0.91 ± 0.08 at S7 vs 0.72 ± 0.11 at pristine stations), demonstrating PARAFAC’s ability to discriminate both natural and anthropogenic DOM sources across the watershed’s land-use gradient.

### 3.3. Variations in fluorescence components

**Fig 4:**
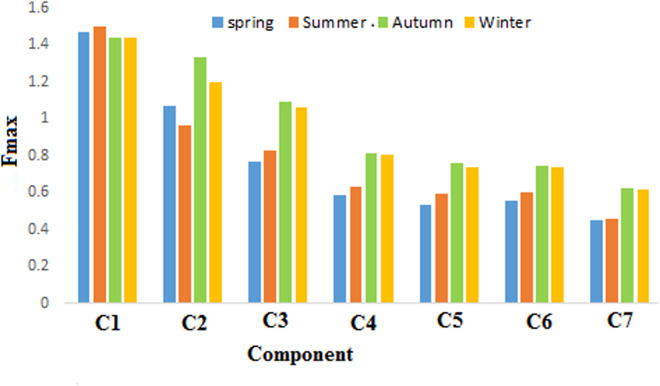
Seasonal variations components in Tajan River.

Fig 4 depicts the average variation of F-max components at different points in each season. The highest F-max of values for components 2, 3, 4, 5, 6, and 7 was identified in autumn, while the minimum f-max for all components was observed in the winter season. Components 3, 4, 5, 6, and 7 exhibited an increasing trend from spring, reaching their peak values in autumn before decreasing in winter. As autumn approached, the number of leaves increased, and organic matter entered the reservoir via surface runoff, leading to a substantial rise in organic matter levels. In winter, the cold temperatures slowed the transformation of organic matters and microorganisms, resulting in minimal changes to the organic matter.

**Fig 5:**
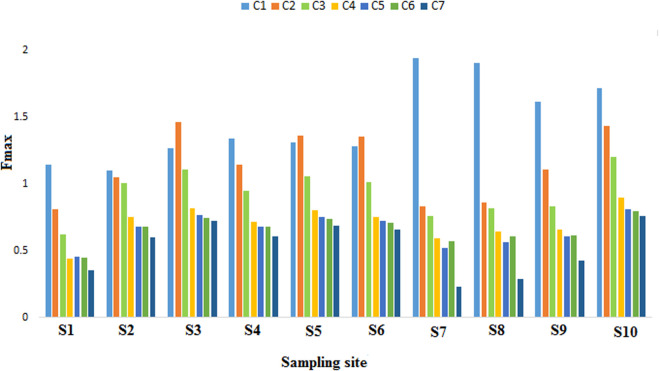
Variations components in Tajan River across 10 sampling points.

In the study by Xu et al. (2021) [19], the components increased from the end of spring to the beginning of summer, peaked in autumn, and then stabilized over the winter. According to the results (Fig 5), the F-max of components 1 and 2 was highest at sampling sites 7 and 3, respectively, and other components peaked at sampling site 10. Humic substances are produced as a result of the hydrolysis of organic residues released into wastewater treatment plants (WWTPs) along with raw, secondary, and tertiary treated wastewater. Additionally, humic substances are formed to a lesser extent through the decomposition of organic matter during biological wastewater treatment and sewage sludge management, particularly during thermal and alkaline pretreatment, as well as in anaerobic digestion [59]. Wastewater from pulp and paper production is loaded with various organic and inorganic pollutants, primarily derived from substances like tannins, lignins, resins, and adsorbed organic compounds [60]. Humic substances form naturally through chemical, physical, and microbial processes that involve the degradation and (re)polymerization of phenolic and aromatic compounds like lignin, tannins, and secondary metabolites [61]. In some estuarine environments, microbial activity may be limited due to factors such as salinity fluctuation [62], low oxygen levels, or the presence of pollutants. This can slow down the degradation of humic material [63].

### 3.4. Fluorescence spectral index

HIX, commonly utilized to assess the humification level of dissolved organic matter (DOM), serves as an indicator of the extent of humification present in aquatic environments. The average values of HIX in all seasons and all sampling points of Tejan River were in the range of 0 to 1.5, which indicates a low degree of humification. The HIX values observed in the Yiluo River watershed varied between 0.12 and 7.85 (mean: 1.57) [64]. Fluorescence Index (FI) serves as an effective tool for determining the sources of humic substances within dissolved organic matter (DOM). FI values in the study area varied between 1.574 and 1.674 in different seasons, this suggests that the origin of humic substances is characterized by a dual contribution, deriving from both terrigenous and endogenous sources. Ma et al. (2022) conducted a study on the Wonggang River and found that the FI values exceeded 1.9 during both the dry and wet seasons. This indicates that microbial metabolism was the primary contributor to the dissolved organic matter (DOM) present in the water [32]. BIX indicates the proportion of autochthonous components within dissolved organic matter (DOM). The value of BIX in Tajan River in spring, autumn, and winter is 0.787, 0.786, and 0.784, respectively, while BIX is 0.800 in summer. Therefore, DOM has moderate autochthonous characteristics in spring, autumn, and winter, and strong autochthonous characteristics in summer. The highest BIX value was observed at sampling site 7 (0.914). Human activities that increase nutrient levels (nitrogen and phosphorus) can enhance primary production and algal metabolism, leading to greater production of biological/microbial dissolved organic matter (DOM) [65]. Chen et al. (2023) reported average BIX values of 1.15 ± 0.16 for the Haihe River and 1.19 ± 0.24 for the Duliujian River (BIX > 1), indicating strong signs of biogenesis [3].

### 3.5. Relationship between Fluorescent Components and Water Quality

**Fig 6:**
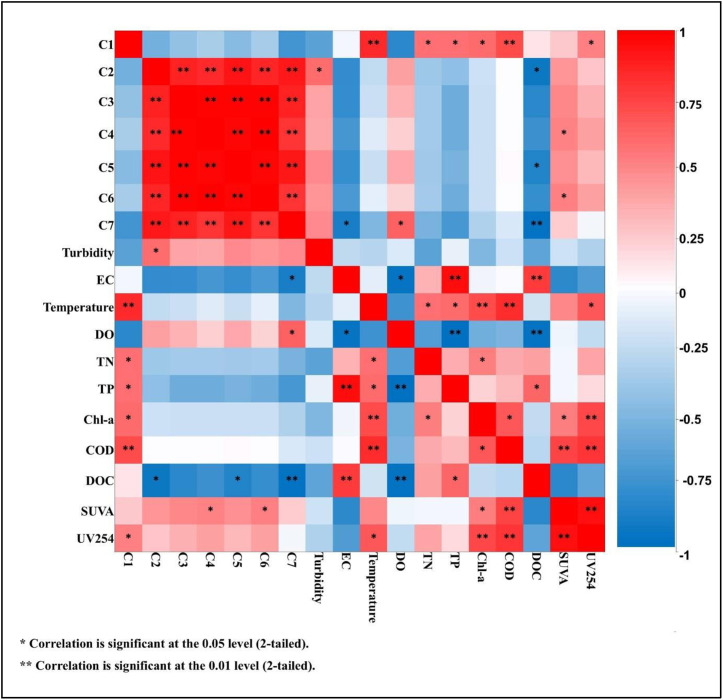
Correlation between Fluorescent Components and Water Quality.

To investigate the correlations between the fluorescent components of dissolved organic matter (C1–C7) and the water quality parameters in the Tajan River, Pearson’s and Spearman’s correlation coefficients were calculated (Fig 6).

C1 exhibited a positive and statistically significant relationship with several wate quality parameters, including temperature (r = 0.858), TN (r = 0.697), TP (r = 0.697), chl-a (r = 0.707), COD (r = 0.794), and UV_254_ absorbance (r = 0.648). This suggest that PARAFAC components, particularly C1, could serve as good indicators of these water quality parameters. Similar significant positive correlations between parameters such as COD, BOD_5_, DO, NH_3_+ -N, TN, pH, TP, Turbidity, and suspended solids, with the fluorescent components (C1,C3 and C4) have been reported in the lakes of the Ebinur region [66]. C2 has a direct relationship with the degree of turbidity and a significant inverse relationship with DOC. A significant positive correlation was identified between terrestrial humic-like components and key indicators such as chlorophyll-a (Chl-a), dissolved reactive phosphorus (DRP), and NH_4_+ -N in the Heilongjiang River [51]. No significant relationship existed between C3 and any of the water quality parameters, but the increase in the SUVA was associated with higher levels of C4 and C6. Component C5 was inversely related to DOC (r = −0.642). C7 had an inverse relationship with DOC (r = −0.769) and EC (r = −0.679), and a significant positive relationship with DO (r = 0.736), while Zhu et al. (2020) reported a weak correlation with EC and tryptophan acid-like components [67].

Our correlation analysis revealed significant land use-DOM linkages, with agricultural areas showing strong positive correlations with humic-like components (C2: r = 0.72; C6: r = 0.69; p < 0.01), reflecting leaching of lignin-rich compounds from croplands. Industrial zones exhibited distinct associations with bulk DOC (r = 0.68) and COD (r = 0.71), indicating wastewater-derived organic matter inputs, while forested areas showed negative correlations with SUVA254 (r = −0.41), suggesting natural vegetation buffers DOM export. These patterns demonstrate how anthropogenic activities alter DOM composition, with agricultural practices enhancing terrestrial humics and urban/industrial development increasing protein-like signals. The stronger agricultural correlations compared to temperate systems (Jones et al., 2020) likely reflect intensive rice cultivation practices in our watershed. These quantitative relationships enable targeted source management, particularly for reducing wastewater-derived DOM in industrialized reaches and optimizing agricultural runoff controls.

## 4. Conclusions

The research focused on examining the compositional variability of dissolved organic matter (DOM) and its relationship to the physical and chemical quality of the Tajan River. The results showed that during the winter season, the river water had lower EC, temperature, turbidity, TP, and SUVA compared to other seasons. In contrast, COD and DOC exhibited the lowest concentration in the spring. The highest TN value and chl-a were observed in the spring. From sampling point 1 to point 10 along the river, the concentration of most of the measured parameters displayed an increasing trend, indicating the impact of various pollutant sources, particularly at station 7 (S7), where the level of EC, temperature, DOC, COD, and TP were higher than other locations. This clearly suggests the influence of the effluent discharge from the Sari city wastewater treatment plant.

The EEM-PARAFAC method was employed to identify the fluorescence components of (DOM). Seven fluorescence component (C1-C7) were identified, where C1 was classified as visible humic-like, C2 and C6 were terrestrial humic- like substances, and C3, C4 and C7 were humic-like substances, likely originated from the discharge of domestic wastewater, agricultural wastewater, and effluents from refineries and various industries. Notably, C1 exhibited a positive and statistically significant relationship with several water quality parameters, including temperature, TN, TP, chl-a, COD and UV_254_ absorbance. This suggests that the PARAFAC components, particularly C1, can serve as of a good indicator of water quality in the Tajan River. The analysis of various fluorescence indices indicated that the humics in dissolved organic matter (DOM) originate from both terrestrial and autochthonous inputs. The observed relationship between the DOM fluorescence component and water quality parameters demonstrate the potential of the EEM-PARAFAC method for monitoring surface water quality, providing valuable insights into the sources and characteristics of DOM in the Tajan River.

## References

[1] Li X-F, Mitch WA. Drinking Water Disinfection Byproducts (DBPs) and Human Health Effects: Multidisciplinary Challenges and Opportunities. Environ Sci Technol. 2018;52(4):1681–9. doi: 10.1021/acs.est.7b05440 29283253

[2] Ikhlaq A, et al. Evaluation of drinking water quality parameters in the areas of East-Lahore Pakistan: A case study. J Faculty Eng Technol (JFET). 2014;21:1–21.

[3] Chen Z, Wen Y, Xiao M, Yue F, Zhang W. Characteristics of Dissolved Organic Matter Impacted by Different Land Use in Haihe River Watershed, China. Int J Environ Res Public Health. 2023;20(3):2432. doi: 10.3390/ijerph20032432 36767800 PMC9915398

[4] Shaheed H, Zawawi MH, Hayder G. The Development of a River Quality Prediction Model That Is Based on the Water Quality Index via Machine Learning: A Review. Processes. 2025;13(3):810. doi: 10.3390/pr13030810

[5] Aazami J, Esmaili-Sari A, Abdoli A, Sohrabi H, Van den Brink PJ. Monitoring and assessment of water health quality in the Tajan River, Iran using physicochemical, fish and macroinvertebrates indices. J Environ Health Sci Eng. 2015;13:29. doi: 10.1186/s40201-015-0186-y 25949817 PMC4422490

[6] Gharibreza M, et al. Assessing the quality of surface sediments in the Tajan River and determining the level of ecological pollution. Environ Water Eng. 2020;6(4):485–500.

[7] Shen W, Zhang L, Ury EA, Li S, Xia B, Basu NB. Restoring small water bodies to improve lake and river water quality in China. Nat Commun. 2025;16(1):294. doi: 10.1038/s41467-024-55714-9 39747225 PMC11697070

[8] Wang W, Zheng B, Jiang X, Chen J, Wang S. Characteristics and Source of Dissolved Organic Matter in Lake Hulun, A Large Shallow Eutrophic Steppe Lake in Northern China. Water. 2020;12(4):953. doi: 10.3390/w12040953

[9] He J, Wu X, Zhi G, Yang Y, Wu L, Zhang Y, et al. Fluorescence characteristics of DOM and its influence on water quality of rivers and lakes in the Dianchi Lake basin. Ecological Indicators. 2022;142:109088. doi: 10.1016/j.ecolind.2022.109088

[10] Zheng Z, et al. Effect of aquatic ecosystem reconstruction on water quality of landscape lake of water supplement purpose by factor analysis. Chinese J Environ Eng. 2014;8(10):4319–25.

[11] Wang X, Wu Y, Bao H, Gan S, Zhang J. Sources, Transport, and Transformation of Dissolved Organic Matter in a Large River System: Illustrated by the Changjiang River, China. J Geophys Res: Biogeosciences. 2019;124(12):3881–901. doi: 10.1029/2018jg004986

[12] Zhang L, Sun Q, Dou Q, Lan S, Peng Y, Yang J. The molecular characteristics of dissolved organic matter in urbanized river sediments and their environmental impact under the action of microorganisms. Sci Total Environ. 2022;827:154289. doi: 10.1016/j.scitotenv.2022.154289 35247414

[13] Mitschke N, Vemulapalli SPB, Dittmar T. NMR spectroscopy of dissolved organic matter: a review. Environ Chem Lett. 2022;21(2):689–723. doi: 10.1007/s10311-022-01528-4

[14] Matilainen A, Gjessing ET, Lahtinen T, Hed L, Bhatnagar A, Sillanpää M. An overview of the methods used in the characterisation of natural organic matter (NOM) in relation to drinking water treatment. Chemosphere. 2011;83(11):1431–42. doi: 10.1016/j.chemosphere.2011.01.018 21316073

[15] Spencer RG, Bolton L, Baker A. Freeze/thaw and pH effects on freshwater dissolved organic matter fluorescence and absorbance properties from a number of UK locations. Water Res. 2007;41(13):2941–50. 17540432 10.1016/j.watres.2007.04.012

[16] Cao T, Li M, Xu C, Song J, Fan X, Li J, et al. Technical note: Chemical composition and source identification of fluorescent components in atmospheric water-soluble brown carbon by excitation–emission matrix spectroscopy with parallel factor analysis – potential limitations and applications. Atmos Chem Phys. 2023;23(4):2613–25. doi: 10.5194/acp-23-2613-2023

[17] Ishii SKL, Boyer TH. Behavior of reoccurring PARAFAC components in fluorescent dissolved organic matter in natural and engineered systems: a critical review. Environ Sci Technol. 2012;46(4):2006–17. doi: 10.1021/es2043504 22280543

[18] Shutova Y, Baker A, Bridgeman J, Henderson RK. Spectroscopic characterisation of dissolved organic matter changes in drinking water treatment: From PARAFAC analysis to online monitoring wavelengths. Water Res. 2014;54:159–69. doi: 10.1016/j.watres.2014.01.053 24568785

[19] Xu X, Kang J, Shen J, Zhao S, Wang B, Zhang X, et al. EEM-PARAFAC characterization of dissolved organic matter and its relationship with disinfection by-products formation potential in drinking water sources of northeastern China. Sci Total Environ. 2021;774:145297. doi: 10.1016/j.scitotenv.2021.145297 33611000

[20] Alahabadi A, Malvandi H. Contamination and ecological risk assessment of heavy metals and metalloids in surface sediments of the Tajan River, Iran. Mar Pollut Bull. 2018;133:741–9. doi: 10.1016/j.marpolbul.2018.06.030 30041371

[21] Sciscenko I, Binetti R, Escudero-Oñate C, Oller I, Arques A. Dissolved Organic Matter Behaviour by Conventional Treatments of a Drinking Water Plant: Controlling Its Changes with EEM-PARAFAC. Applied Sciences. 2024;14(6):2462. doi: 10.3390/app14062462

[22] Capowiez Y, Sanchez-Hernandez JC. Anecic earthworms benefit from organic management practices in apple orchards under the Mediterranean climate. Applied Soil Ecol. 2024;200:105439. doi: 10.1016/j.apsoil.2024.105439

[23] Sharifinia M, Imanpour Namin J, Bozorgi Makrani A. Benthic macroinvertabrate distribution in Tajan River using canonical correspondence analysis. 2012.

[24] Zazouli M, et al. Temporal and spatial variation of nitrate and nitrite concentration in drinking water resource in Kohgiluyeh county using geographic information system. Jf Mazandaran University Med Sci. 2014;23(109):258–63.

[25] Wilson TP, Miller CV, Lechner EA. Guidelines for the use of automatic samplers in collecting surface-water quality and sediment data. US Geological Survey. 2024.

[26] Lipps WC, B EBB-H, Baxter TE. Standard Methods for the Examination of Water and Wastewater. 24th ed. 2023.

[27] Luo Y, Zhang Y, Lang M, Guo X, Xia T, Wang T, et al. Identification of sources, characteristics and photochemical transformations of dissolved organic matter with EEM-PARAFAC in the Wei River of China. Front Environ Sci Eng. 2021;15(5). doi: 10.1007/s11783-020-1340-z

[28] Bro R. Parafac. Tutorial and applications. Chemomet intelligent laboratory syst. 1997;38(2):149–71.

[29] Zhang S, Bai Y, Wen X, Ding A, Zhi J. Seasonal and downstream alterations of dissolved organic matter and dissolved inorganic ions in a human-impacted mountainous tributary of the Yellow River, China. Environ Sci Pollut Res Int. 2018;25(18):17967–79. doi: 10.1007/s11356-018-1972-8 29680893

[30] Zhang Y, Zhang E, Yin Y, van Dijk MA, Feng L, Shi Z, et al. Characteristics and sources of chromophoric dissolved organic matter in lakes of the Yungui Plateau, China, differing in trophic state and altitude. Limnol Oceanogr. 2010;55(6):2645–59. doi: 10.4319/lo.2010.55.6.2645

[31] Tang J, Li X, Cao C, Lin M, Qiu Q, Xu Y, et al. Compositional variety of dissolved organic matter and its correlation with water quality in peri-urban and urban river watersheds. Ecological Indicators. 2019;104:459–69. doi: 10.1016/j.ecolind.2019.05.025

[32] Ma J, Pei D, Zhang X, Lai Q, He F, Fu C, et al. The Distribution of DOM in the Wanggang River Flowing into the East China Sea. Int J Environ Res Public Health. 2022;19(15):9219. doi: 10.3390/ijerph19159219 35954582 PMC9367814

[33] Cory RM, McKnight DM. Fluorescence spectroscopy reveals ubiquitous presence of oxidized and reduced quinones in dissolved organic matter. Environ Sci Technol. 2005;39(21):8142–9. doi: 10.1021/es0506962 16294847

[34] Zheng S, Wang P, Wang C, Hou J, Qian J. Distribution of metals in water and suspended particulate matter during the resuspension processes in Taihu Lake sediment, China. Quaternary Int. 2013;286:94–102. doi: 10.1016/j.quaint.2012.09.003

[35] Zhang L, Zhang L, Zhang D, Cen Y, Wang S, Zhang Y, et al. Analysis of Seasonal Water Characteristics and Water Quality Responses to the Land Use/Land Cover Pattern: A Case Study in Tianjin, China. Water. 2023;15(5):867. doi: 10.3390/w15050867

[36] Wen Y, Xiao M, Chen Z, Zhang W, Yue F. Seasonal Variations of Dissolved Organic Matter in Urban Rivers of Northern China. Land. 2023;12(2):273. doi: 10.3390/land12020273

[37] Yasmin F, Hossain T, Shahrukh S, Hossain ME, Sultana GNN. Evaluation of seasonal changes in physicochemical and bacteriological parameters of Gomti River in Bangladesh. Environ Sustain Indicat. 2023;17:100224. doi: 10.1016/j.indic.2023.100224

[38] Pakoksung K, Inseeyong N, Chawaloesphonsiya N, Punyapalakul P, Chaiwiwatworakul P, Xu M, et al. Seasonal dynamics of water quality in response to land use changes in the Chi and Mun River Basins Thailand. Sci Rep. 2025;15(1):7101. doi: 10.1038/s41598-025-91820-4 40016376 PMC11868589

[39] Majlesi M, Alavi N, Atamaleki A, Seyyed M, Soheili N. Investigation of Physicochemical and Microbial Parameters of Doogh River Water and the Effect of Adjacent Felman Wells on Decreasing These Parameters in Kalaleh city During 2012-2016. J Environ Health Eng. 2019;7(1):29–41. doi: 10.29252/jehe.7.1.29

[40] Wang X, Zhao L, Xu H, Zhang X. Spatial and seasonal characteristics of dissolved heavy metals in the surface seawater of the Yellow River Estuary, China. Mar Pollut Bull. 2018;137:465–73. doi: 10.1016/j.marpolbul.2018.10.052 30503457

[41] Deng J, Chen F, Hu W, Lu X, Xu B, Hamilton DP. Variations in the Distribution of Chl-a and Simulation Using a Multiple Regression Model. Int J Environ Res Public Health. 2019;16(22):4553. doi: 10.3390/ijerph16224553 31752099 PMC6888353

[42] Winder M, Cloern JE. The annual cycles of phytoplankton biomass. Philos Trans R Soc Lond B Biol Sci. 2010;365(1555):3215–26. doi: 10.1098/rstb.2010.0125 20819814 PMC2981943

[43] Buchan A, LeCleir GR, Gulvik CA, González JM. Master recyclers: features and functions of bacteria associated with phytoplankton blooms. Nat Rev Microbiol. 2014;12(10):686–98. doi: 10.1038/nrmicro3326 25134618

[44] del Barrio P, Ganju NK, Aretxabaleta AL, Hayn M, García A, Howarth RW. Modeling future scenarios of light attenuation and potential seagrass success in a eutrophic estuary. Estuarine, Coastal and Shelf Science. 2014;149:13–23. doi: 10.1016/j.ecss.2014.07.005

[45] Lu Z, Gan J. Controls of seasonal variability of phytoplankton blooms in the Pearl River Estuary. Deep Sea Research Part II: Topical Studies in Oceanography. 2015;117:86–96. doi: 10.1016/j.dsr2.2013.12.011

[46] Islam ST, et al. Comprehensive assessment of trophic status and chlorophyll-a dynamics in the Jhelum River Basin: Implications for river ecosystem management. Int J Environ Res. 2025;19(2):48.

[47] Varol M. Spatio-temporal changes in surface water quality and sediment phosphorus content of a large reservoir in Turkey. Environ Pollut. 2020;259:113860. doi: 10.1016/j.envpol.2019.113860 31887594

[48] De Borba BM, Jack RF, Rohrer JS, Wirt J, Wang D. Simultaneous determination of total nitrogen and total phosphorus in environmental waters using alkaline persulfate digestion and ion chromatography. J Chromatogr A. 2014;1369:131–7. doi: 10.1016/j.chroma.2014.10.027 25441080

[49] Li T, Zhou P, Ding Y, Tang Q, Zhou S, Liu Y. Distribution Characteristics and Source Analysis of Nitrogen and Phosphorus in Different Rivers in Two Water Period: A Case Study of Pi River and Shiting River in the Upper Reaches of Tuo River in China. Int J Environ Res Public Health. 2022;19(19):12433. doi: 10.3390/ijerph191912433 36231734 PMC9566003

[50] Li J, Luo G, He L, Xu J, Lyu J. Analytical Approaches for Determining Chemical Oxygen Demand in Water Bodies: A Review. Crit Rev Anal Chem. 2018;48(1):47–65. doi: 10.1080/10408347.2017.1370670 28857621

[51] Cui H, Shi J, Qiu L, Zhao Y, Wei Z, Wang X, et al. Characterization of chromophoric dissolved organic matter and relationships among PARAFAC components and water quality parameters in Heilongjiang, China. Environ Sci Pollut Res Int. 2016;23(10):10058–71. doi: 10.1007/s11356-016-6230-3 26865492

[52] Haaland S, Hejzlar J, Eikebrokk B, Orderud G, Paule-Mercado MaC, Porcal P, et al. Predicting the dissolved natural organic matter (DNOM) concentration and the specific ultraviolet absorption (sUVa) index in a browning central European stream. Ecological Indicators. 2024;165:112200. doi: 10.1016/j.ecolind.2024.112200

[53] Ma Q, Li G, Wei Y. Spectral characteristics and spatiotemporal variation of DOM in peri-urban critical zone. Environ Chem. 2020;2:455–66.

[54] Shi W, Zhuang W-E, Hur J, Yang L. Monitoring dissolved organic matter in wastewater and drinking water treatments using spectroscopic analysis and ultra-high resolution mass spectrometry. Water Res. 2021;188:116406. doi: 10.1016/j.watres.2020.116406 33010601

[55] Liu Q, Jiang Y, Tian Y, Hou Z, He K, Fu L, et al. Impact of land use on the DOM composition in different seasons in a subtropical river flowing through a region undergoing rapid urbanization. J Cleaner Prod. 2019;212:1224–31. doi: 10.1016/j.jclepro.2018.12.030

[56] Yang L, Cheng Q, Zhuang W-E, Wang H, Chen W. Seasonal changes in the chemical composition and reactivity of dissolved organic matter at the land-ocean interface of a subtropical river. Environ Sci Pollut Res Int. 2019;26(24):24595–608. doi: 10.1007/s11356-019-05700-2 31236861

[57] Zhang Y, Yin Y, Feng L, Zhu G, Shi Z, Liu X, et al. Characterizing chromophoric dissolved organic matter in Lake Tianmuhu and its catchment basin using excitation-emission matrix fluorescence and parallel factor analysis. Water Res. 2011;45(16):5110–22. doi: 10.1016/j.watres.2011.07.014 21840562

[58] Zhang H, Cui K, Guo Z, Li X, Chen J, Qi Z, et al. Spatiotemporal variations of spectral characteristics of dissolved organic matter in river flowing into a key drinking water source in China. Sci Total Environ. 2020;700:134360. doi: 10.1016/j.scitotenv.2019.134360 31629259

[59] Michalska J, Turek-Szytow J, Dudło A, Surmacz-Górska J. Characterization of humic substances recovered from the sewage sludge and validity of their removal from this waste. EFB Bioeconomy Journal. 2022;2:100026. doi: 10.1016/j.bioeco.2022.100026

[60] Ashrafi O, Yerushalmi L, Haghighat F. Wastewater treatment in the pulp-and-paper industry: A review of treatment processes and the associated greenhouse gas emission. J Environ Manage. 2015;158:146–57. doi: 10.1016/j.jenvman.2015.05.010 25982876

[61] Kulikova NA, Perminova IV. Interactions between Humic Substances and Microorganisms and Their Implications for Nature-like Bioremediation Technologies. Molecules. 2021;26(9):2706. doi: 10.3390/molecules26092706 34063010 PMC8124324

[62] Zhang G, Bai J, Tebbe CC, Zhao Q, Jia J, Wang W, et al. Salinity controls soil microbial community structure and function in coastal estuarine wetlands. Environ Microbiol. 2021;23(2):1020–37. doi: 10.1111/1462-2920.15281 33073448

[63] Wang H, Chen F, Zhang C, Wang M, Kan J. Estuarine gradients dictate spatiotemporal variations of microbiome networks in the Chesapeake Bay. Environ Microbiome. 2021;16(1):22. doi: 10.1186/s40793-021-00392-z 34838139 PMC8627074

[64] Shao T, Wang T. Effects of land use on the characteristics and composition of fluvial chromophoric dissolved organic matter (CDOM) in the Yiluo River watershed, China. Ecological Indicators. 2020;114:106332. doi: 10.1016/j.ecolind.2020.106332

[65] Tang J, Wang W, Yang L, Qiu Q, Lin M, Cao C, et al. Seasonal variation and ecological risk assessment of dissolved organic matter in a peri-urban critical zone observatory watershed. Sci Total Environ. 2020;707:136093. doi: 10.1016/j.scitotenv.2019.136093 31863979

[66] Wang X, Zhang F, Kung H, Ghulam A, Trumbo AL, Yang J, et al. Evaluation and estimation of surface water quality in an arid region based on EEM-PARAFAC and 3D fluorescence spectral index: A case study of the Ebinur Lake Watershed, China. CATENA. 2017;155:62–74. doi: 10.1016/j.catena.2017.03.006

[67] Zhu G, Xiong N, Wang X, Hursthouse AS, Marr A. Correlation Characteristics of Electrical Conductivity of Surface Waters with the Fluorescence Excitation-Emission Matrix Spectroscopy-Parallel Factor Components of Dissolved Organic Matter. J Fluoresc. 2020;30(6):1383–96. doi: 10.1007/s10895-020-02628-6 32997315

